# The ADVanced Organ Support (ADVOS) hemodialysis system removes IL-6: an in vitro proof-of-concept study

**DOI:** 10.1186/s40635-024-00652-5

**Published:** 2024-07-31

**Authors:** Susanne Himmelein, Aritz Perez Ruiz de Garibay, Veronika Brandel, Frank Zierfuß, Tobias Michael Bingold

**Affiliations:** 1 ADVITOS GmbH, Munich, Germany; 2 Ostbayerische Technische Hochschule Regensburg, Regensburg, Germany; 3 Department of Internal Medicine, Universitätsklinikum Frankfurt, Frankfurt, Germany

**Keywords:** Extracorporeal blood purification, Albumin dialysis, Multiple organ failure, Advanced organ support, IL-6, Cytokines

## Abstract

**Background:**

IL-6 is a pleiotropic cytokine modulating inflammation and metabolic pathways. Its proinflammatory effect plays a significant role in organ failure pathogenesis, commonly elevated in systemic inflammatory conditions. Extracorporeal blood purification devices, such as the Advanced Organ Support (ADVOS) multi hemodialysis system, might offer potential in mitigating IL-6's detrimental effects, yet its efficacy remains unreported.

**Methods:**

We conducted a proof-of-concept in vitro study to assess the ADVOS multi system's efficacy in eliminating IL-6. Varying concentrations of IL-6 were introduced into a swine blood model and treated with ADVOS multi for up to 12 h, employing different blood and concentrate flow rates. IL-6 reduction rate, clearance, and dynamics in blood and dialysate were analyzed.

**Results:**

IL-6 clearance rates of 0.70 L/h and 0.42 L/h were observed in 4 and 12-h experiments, respectively. No significant differences were noted across different initial concentrations. Reduction rates ranged between 40 and 46% within the first 4 h, increasing up to 72% over 12 h, with minimal impact from flow rate variations. Our findings suggest that an IL-6-albumin interaction and convective filtration are implicated in in vitro IL-6 elimination with ADVOS multi.

**Conclusions:**

This study demonstrates for the first time an efficient and continuous in vitro removal of IL-6 by ADVOS multi at low blood flow rates. Initial concentration-dependent removal transitions to more consistent elimination over time. Further clinical investigations are imperative for comprehensive data acquisition.

## Background

Critical care medicine has witnessed remarkable advancements in recent years, yet the management of critically ill patients with organ failures remains a formidable challenge. Among the complex web of inflammatory mediators orchestrating the systemic inflammatory response in these patients, interleukin-6 (IL-6) has garnered significant attention for its potential role in exacerbating organ dysfunction and adversely affecting clinical outcomes. In this context, extracorporeal blood purification devices, have emerged as innovative tools with the potential to mitigate the detrimental effects of excessive IL-6 [1].

IL-6 is a pleiotropic cytokine affecting inflammation and metabolic pathways. While IL-6 can exert positive effects, such as promoting an anti-inflammatory state of macrophages, limiting atheroma formation, or mediating insulin-sensitizing effects of physical exercise [2], its recent therapeutic interest is based on its negative effects. In its proinflammatory role, IL-6 is known to contribute to the pathogenesis of organ failure, coagulopathy, and immune dysregulation in critically ill patients and has been shown to be elevated in various acute systemic inflammatory syndromes and secondary organ dysfunctions (i.e., renal, hepatic or pulmonary) [3]. Increased levels of IL-6 induce the expression of various genes involved in inflammation, cell survival, and differentiation, which might result in the promotion of fever, acute phase response, endothelial activation, coagulation dysfunction, tissue injury, and organ dysfunction [3]. This makes IL-6 an attractive target for therapeutic intervention.

In this regard, different therapeutic strategies may be used, such as corticosteroids, antiviral agents, anti-cytokine antibodies or inhibitors (e.g., tocilizumab, anakinra, sarilumab), immunomodulators (e.g., baricitinib, ruxolitinib), convalescent plasma therapy, and extracorporeal blood purification (EBP) [4]. However, a significant knowledge gap exists concerning the direct elimination of IL-6 from the bloodstream using EBP. While several cytokine adsorption devices have reported IL-6 removal, the clinical efficacy of these interventions is being currently debated [5, 6]. Moreover, very few studies exist on the reduction of IL-6 levels using EBP systems that do not incorporate adsorptive materials or devices.

Among the plethora of therapies for critically ill patients, the ADVOS multi hemodialysis system has emerged as a promising medical device for the support of the liver, the kidney and the lung [7]. Based on the principle of albumin dialysis and using a customizable recirculating dialysate, ADVOS has reported the removal of protein-bound and water-soluble toxins while allowing a fluid-based CO_2_ removal and the correction of acid–base balance [8–15]. Understanding the potential of this device to remove IL-6 could represent a groundbreaking advancement in the field of critical care medicine, offering a novel approach to ameliorate the systemic inflammatory response associated with organ failure.

The primary aim of this study is to bridge the existing knowledge gap by investigating the efficacy of the ADVOS multi system in eliminating IL-6 from human blood in an in vitro setting. Our research seeks to determine the IL-6 removal kinetics of the ADVOS multi system. For this purpose, several scenarios using various IL-6 concentrations were tested. By comprehensively addressing these questions, we aim to contribute to a deeper understanding of the potential role of the ADVOS therapy in targeting IL-6, ultimately paving the way for novel therapeutic strategies that may enhance the management and outcomes of critically ill patients with organ failure.

## Methods

### Blood model

Fresh porcine blood was sourced from a local slaughterhouse (Münchner Schlachthof Betriebs GmbH, Munich, Germany) and processed following a standardized protocol. The blood was mixed with a modified Ringer's solution to attain a hematocrit level of 36%, maintaining standard electrolyte concentrations and typical blood gas values. To prevent coagulation, we administered 30,000 IU of heparin per liter of blood (Ratiopharm, Ulm, Germany). The blood was maintained at a constant temperature of 37 °C and gently agitated at a rate of 130 rpm to ensure its suitability for our intensive care research.

Different concentrations of IL-6 were spiked in blood. Briefly, recombinant human IL-6 (PeproTech, Cranbury, NJ, USA) was prepared according to the manufacturer's instructions for handling and reconstitution. The IL-6 was initially reconstituted in distilled water by gently shaking the vial after centrifugation. The IL-6 reconstituted solution was stored at 2 °C for short term use (up to 1 week) or at − 20 °C to ensure long term stability. During experiments, 0.5, 2.5, 10 and 50 µg of IL-6 were spiked into 5 L blood to achieve IL-6 concentrations of 100, 500, 2000 and 10,000 pg/mL, respectively.

### ADVOS multi-hemodialysis system

**Fig. 1:**
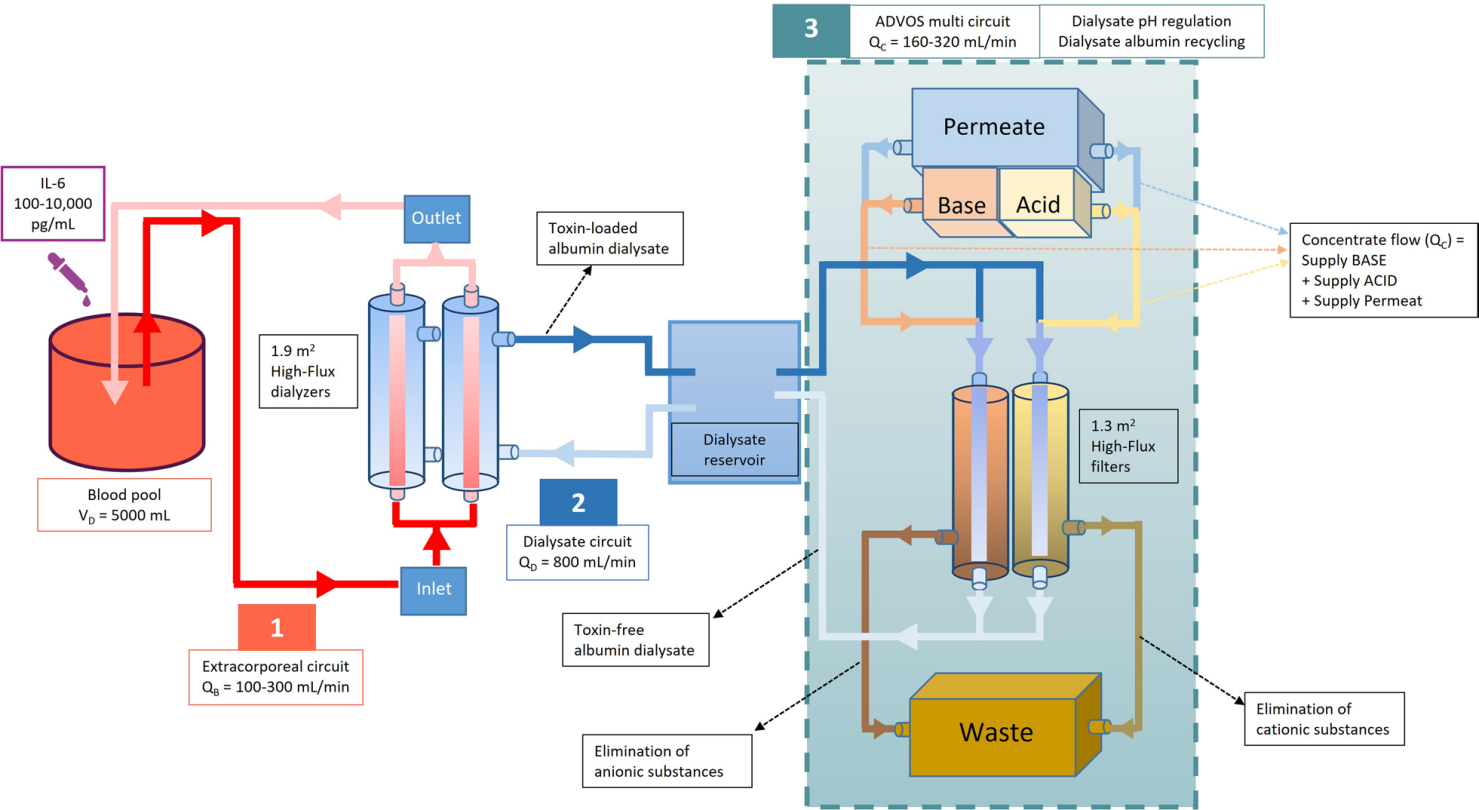
Schematic representation of ADVOS multi and the experimental setting. A blood pool of 5 L was spiked with different concentrations of IL-6 and was subsequently treated with ADVOS multi. The ADVOS hemodialysis system consists of an extracorporeal circuit, a dialysate circuit and an albumin regeneration circuit (i.e., ADVOS multi circuit)

The ADVOS multi (ADVITOS GmbH, Munich, Germany) is a hemodialysis system intended for the removal of water-soluble and protein-bound substances, for the correction of blood composition in case of electrolyte imbalance, including metabolic or hypercapnic acidosis, and for the removal of fluid, if needed. It consists of three interconnected circuits (Fig. 1). Briefly, bloods flows (100–400 mL/min) through the extracorporeal circuit, which bears two ELISIO 19H dialyzers (Nipro D.Med Germany GmbH, Hamburg, Germany). Here, human albumin enriched (200 mL, 20%) dialysate flowing from the dialysate circuit (800 mL/min) receives toxins from blood. The toxin-loaded dialysate fluid enters and recirculates then through the ADVOS multi regeneration circuit. Here, by applying pH and temperature changes, dialysate albumin adapts its structure to release the protein-bound toxins. These are then filtered by convection via two ELISIO 13H filters (Nipro D. Med Germany GmbH, Hamburg, Germany) into the waste together with the water-soluble toxins. The removed volume is replenished with fresh dialysate (160–320 mL/min), which is obtained by continuously mixing osmosis water, an alkaline (i.e., mainly NaOH), and an acidic concentrate (i.e., mainly HCl) online. Finally, the acidic to alkaline concentrate ratio defines the individualized dialysate pH (7.2–9.5).

Additionally, in order to maintain adequate and physiological blood gas values, CO_2_ was continuously infused into the blood pool via an additional ELISIO 19H dialyzer (Nipro D.Med Germany GmbH, Hamburg, Germany) connected to a CO_2_ gas supply (Linde AG, Munich, Germany), as previously described [13].

### Experimental set-up

In a preliminary analysis to rule out a spontaneous change of IL-6 levels, blood was spiked with a known concentration of IL-6. Blood was continuously stirred for 4 h without further treatment. Duplicate samples were taken and analyzed at different timepoints. Blood without IL-6 addition was used as negative control.

Once stated that no decomposition of IL-6 occurred in our blood model, blood was subjected to 4-h treatments with ADVOS multi. Table 1 shows the different settings that were employed with the aim to determine the effect of varying IL-6 concentrations, and blood and concentrate flows on the reduction rate and clearance of IL-6. Finally, to state if IL-6 could be continuously removed beyond 4 h, treatments lasting 12 h were conducted with a starting concentration of 10,000 pg/mL IL-6.

**Table 1:** Experimental settings during ADVOS multi treatments

IL-6 (pg/mL)	100	500	2000	10,000	10,000	10,000	10,000
Blood flow (mL/min)	100	100	100	100	300	100	300
Concentrate flow (mL/min)	160	160	160	160	320	160	320
Dialysate flow (mL/min)	800	800	800	800	800	800	800
Dialysate pH	7.8	7.8	7.8	7.8	7.8	7.8	7.8
Duration (h)	4	4	4	4	4	12	12

Each experiment was conducted three times

Blood samples were taken in duplicate at minutes 0, 15, 30, 60, 120, 180, and 240 (4 h), and additionally at minutes 480, 720 (12 h) for the longer experiments. Each experiment was performed 3 times.

### IL-6 measurement and removal analysis

Blood and dialysate samples (5 mL) were obtained at the specified time points. Blood was centrifuged at 4000 rpm for 15 min at 4 °C and 1 mL of the blood plasma was stored at − 25 °C until analysis. Dialysate did not undergo centrifugation. The Elecsys IL-6 sandwich chemiluminescent immunoassay (Roche Diagnostics GmbH, Mannheim, Germany) was used for sample analysis. Briefly, a sample of 18 µL is initially mixed with IL-6 specific antibodies and then incubated with ruthenium labelled IL-6 specific antibodies to form a sandwich complex. The complexes are then magnetically captured, inducing a chemiluminescent emission proportional to IL-6 concentration.

#### Reduction rate and clearance

Reduction rate was calculated as stated in Eq. 1, where C_f_ refers to the final IL-6 concentration (e.g., at 240 or 720 min for 4 and 12 h experiments, respectively), and *C*_0_ is the started spiked IL-6 concentration.

1 $$\text{Reduction rate} \left(\%\right)=1-\frac{{C}_{f}}{{C}_{0}} \times 100$$

Equation 2 was employed for the calculation of the elimination constant (*K*_e_), where *t*_f_ and *t*_0_ reflect the final (i.e., 4 or 12 h) and the starting (i.e., 0 min) timepoints, respectively. *K*_e_ was calculated assuming a first order kinetics.

2 $${K}_{e}({h}^{-1})=\frac{\text{ln}(\frac{{C}_{0}}{{C}_{f}})}{({t}_{f}-{t}_{0})}=\frac{\text{ln}{C}_{0}-\text{ln}{C}_{f}}{({t}_{2}-{t}_{1})}$$

For clearance (CL) calculation, a volume of distribution (Vd) of 5 L (i.e., blood volume) was set within Eq. 3.

3 $$\text{CL} (l/h)=\text{Vd}\times {K}_{e}$$

### Statistical analysis

Data were documented and analyzed using Microsoft Excel and IBM SPSS 28.0 for Windows®, respectively. Reduction rate, and clearance are presented as mean and standard deviation (SD). An analysis of variance (ANOVA) followed by Bonferroni tests was conducted to determine differences between varying treatment settings. A *p* value lower than 0.05 was considered to indicate statistical significance.

## Results

In the preliminary test, IL-6 was shown to be stable in the blood model with a statistically non-significant mean level change of 5% (data not shown). No IL-6 was detected in the negative control.

**Fig. 2:**
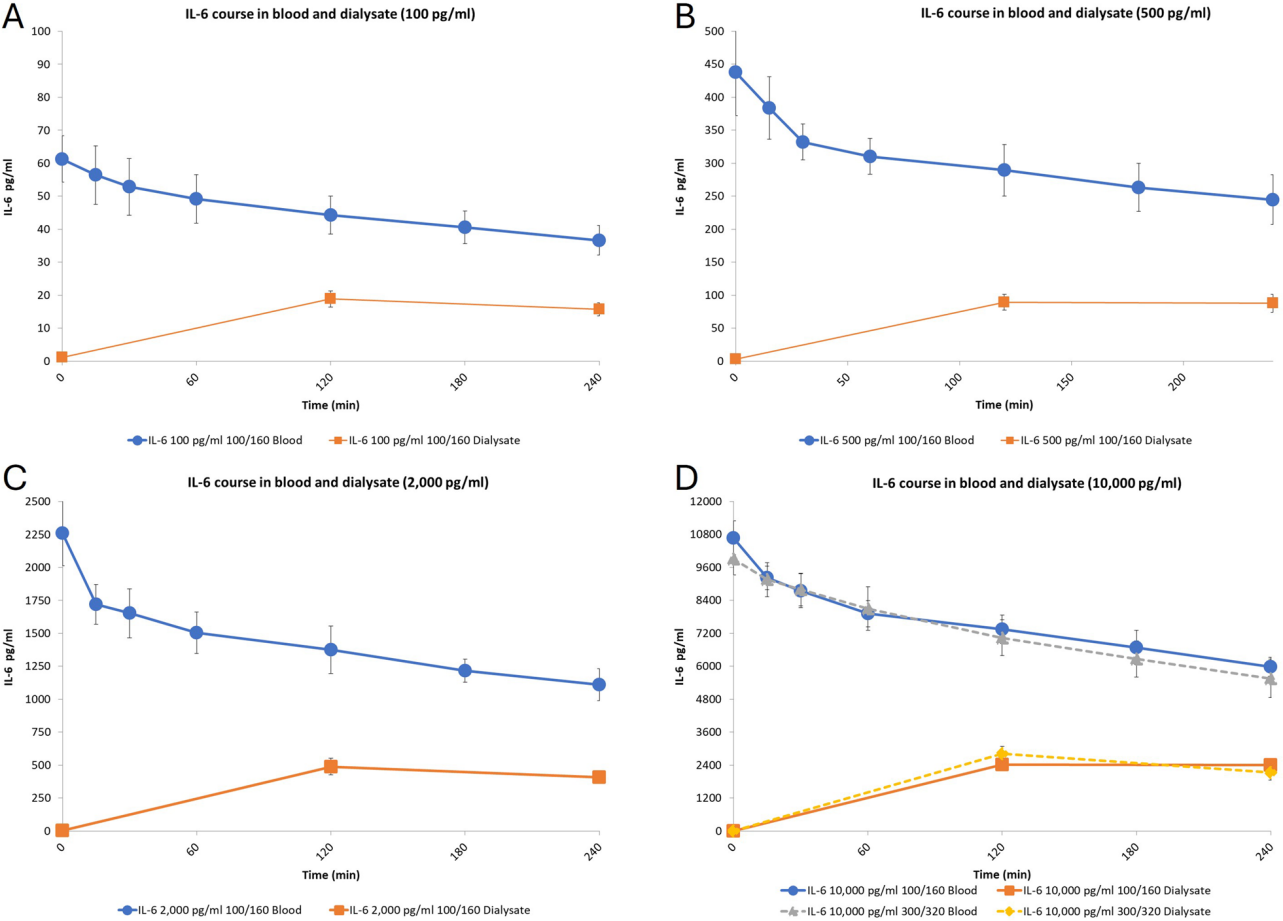
Course of IL-6 in blood (blue lines) and dialysate (orange lines) during 4 h treatment with ADVOS multi at varying starting concentrations. **A** 100 pg/mL; **B** 500 pg/mL; **C** 2,000 pg/mL; **D** 10,000 pg/mL. Error bars represent SD

During treatments with ADVOS multi, a continuous removal of IL-6 was demonstrated. For all the settings tested, the course of IL-6 in blood experienced a fast decrease during the first 30 min and then continued to be removed in a more stable manner for the whole observational period (Fig. 2). Concomitantly, dialysate IL-6 levels increased first to then correlate with the decrease of IL-6 observed in blood (Fig. 2).

**Fig. 3:**
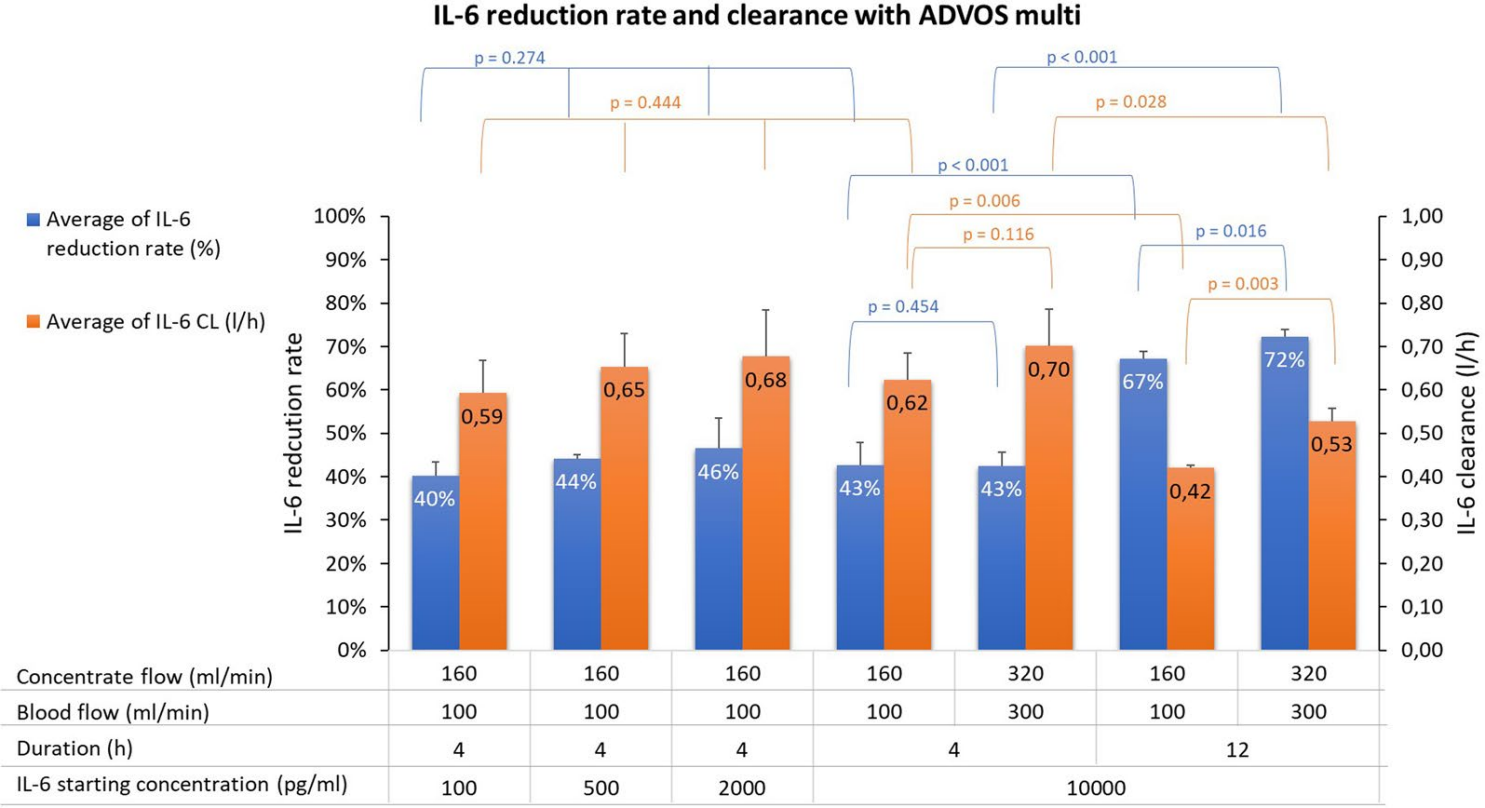
IL-6 reduction rate (blue columns) and clearance (orange columns) mean values for the different settings tested during ADVOS multi treatments. Error bars represent SD. Significance levels obtained by ANOVA are shown for each pair/group

The mean clearance of IL-6 with ADVOS multi varied between 0.42 L/h and 0.70 L/h (Fig. 3, orange columns). No significant differences between 4 h experiments at varying starting concentrations (*p* = 0.444) were detected. A statistical difference between different flow combinations was only detected in 12 h experiments (0.42 vs. 0.53 L/h, *p* = 0.003), but not in 4 h experiments (0.62 vs 0.70 l/h, *p* = 0.116) with blood flow 100 mL/min combined with concentrate flow 160 mL/min, and with blood flow 300 mL/min combined with concentrate flow 320 mL/min, respectively.

The calculated clearance resulted in a mean reduction rate of IL-6 with ADVOS multi between 40 and 46% for the first 4 h and up to 72% for 12 h treatments (Fig. 3, blue columns). No significant statistical differences were observed among the varying concentrations tested (*p* = 0.274). The increase of blood flow and concentrate flow did not significantly change the removal rate of IL-6 in the 4 h treatment (43% vs. 43%, *p* = 0.454) but was statistically significant in 12 h treatments (67% vs. 72%, *p* = 0.016).

## Discussion

### Key results

This proof-of-concept work shows for the first time that the treatment with ADVOS multi can efficiently and continuously remove IL-6 from blood in an in vitro setting. ADVOS multi was able to remove up to 72% of the spiked IL-6 during 12 h of treatment. Moreover, removal rate was comparable for all the starting concentrations tested (100–10,000 pg/mL) and increasing blood and concentrate flows barely improved IL-6 elimination.

### Interpretation

The ADVOS hemodialysis system is intended for patients with multiple organ failure suffering from acute, chronic and acute-on-chronic liver failure and/or renal failure and/or acidosis [7]. In these patients, especially in the presence of acidosis [16], a hyperinflammatory state with elevated levels of IL-6 might be present [17–20]. The mitigation of the hyperinflammation through the removal of IL-6 has been suggested as a therapeutic option [21].

IL-6 is a middle-molecule of about 24.5 kDa. Due to its size, it is not expected to be removed through conventional high-flux dialyzers [22]. ADVOS multi uses two polyethersulfone high flux hemodialyzers accounting for a surface of 3.8 m^2^ (i.e., 2 × 1.9 m^2^) in the extracorporeal circuit. In a hemodialysis setting, these filters have shown a good removal rate for small molecules [23], but a limited removal of large to medium molecules is expected [24, 25], especially at molecular weights above 20 kDa [23]. Even if limited through its size, one could expect that at higher initial IL-6 concentrations, higher gradients and consequently higher reductions rates could be achieved, attending to Fick’s Law [26]. This would be true for a diffusion-driven-elimination. However, the degree of elimination is not solely determined by the cytokine's molecular weight; instead, it's influenced by factors like charge, hydrophilicity, or carrier binding [27]. As demonstrated by other authors, cytokine removal is not only a diffusive process, and convection, and/or adsorption need to be considered [28]. In our experiments, we did not use any adsorptive membranes and changes in blood flow or initial concentrations did not exert any influence on the IL-6 reduction rate with ADVOS (Fig. 3). This points towards additional elimination mechanisms.

In this regard, an albumin-mediated IL-6 removal has been hypothesized based on a demonstrated in vitro interaction between albumin and IL-6 [29]. The authors of the study suggest that the presence of albumin within the dialysate facilitates an increased passage of cytokines over membrane pores and increases the affinity for albumin-associated cytokines on the dialysate side.

On top of a possible impact of dialysate albumin, convective transport might also play a role. ADVOS multi is a hemodialysis system and diffusive solute removal is, therefore, expected in the extracorporeal circuit. In addition to this, in the dialysate regeneration circuit, convective filtration occurs through two high flux filters of 1.3 m^2^ each. Concentrate flow refers to the amount of dialysate that is filtered and replaced. In detail, the amount of new dialysate entering the circuit accounts for the sum of volumes/flows of fresh permeate supply, of new BASE concentrate supply and of new ACID concentrate supply. This volume of fresh dialysate is compensated with the same volume of dialysate exiting the circuit into the waste at the same flow (i.e., concentrate flow). In our work, concentrate flows of 160 and 320 mL/min were tested, which could have been responsible for convective removal. Glancey et al. reported this phenomenon as increasing values of ultrafiltration during hemodiafiltration with high flux filters resulted in higher removal rates for low molecular weight proteins, including ß2-Microglobulin, Myoglobin and IL-6 [30].

The role of the dialysate regeneration circuit in ADVOS multi has additional particularities. As described above, the toxin-loaded dialysate enters the regeneration circuit where dialysate albumin unloading occurs and both protein-bound and water-soluble toxins are filtered by convection in exchange for fresh dialysate. However, a small proportion of dialysate does not enter this circuit, which allows to maintain a significant level of dialysate constituents. This works as a safety mechanism to avoid an excessive loss of relevant substances. Nevertheless, it allows a sufficient concentration gradient for an efficient continuous removal, as shown in Fig. 2. Although this might be seen as a disadvantage in comparison to conventional single-pass systems, it has been shown to be important to avoid the loss of anti-inflammatory cytokines [31].

Indeed, this work by Kaps et al. was the first to report data on cytokine removal with ADVOS multi [31]. The authors analyzed a panel of pro- and anti-inflammatory cytokines before and after a single ADVOS treatment in patients with acute-on-chronic liver failure (ACLF) and concluded that concentrations of pathomechanistically relevant cytokines remained unchanged. On one hand, the authors did not analyze levels before and after the dialyzers. Therefore, removal of interleukins cannot be ruled out, even if systemic plasma levels did not significantly change before and after treatment. An absence of correlation between IL-6 removal rate and reduction of systemic levels has already been documented for other blood purification devices [32]. On the other hand, the recirculation occurring within the ADVOS multi device could hinder an excessive removal of cytokines, when levels are close to physiologic values, which can be as high as 43.5 pg/mL for IL-6, as documented in a recent meta-analysis [33].

### Generalizability

Attempts to reduce the systemic levels of IL-6 by other blood purification systems in a clinical setting has largely been documented [6, 34]. However, the focus on middle to large molecule removal is based either on adsorptive devices with the highest reduction rates in vitro [28], or membranes with high- and medium-cut-off (HCO and MCO, respectively) [23–25, 35–37]. When utilizing HCO or MCO membranes for convective therapy, it's essential to prevent significant albumin leakage during a renal replacement therapy session. While increasing the convection volume enhances middle molecule removal, a larger volume also carries a higher risk of albumin leakage [38].

Our work shows that ADVOS multi can remove IL-6 at low blood flows using conventional high flux filters, which minimizes albumin loss. Being this said, the main goal of the ADVOS therapy is not the sole removal of IL-6, for which other devices such as HCO or adsorptive membranes have been specifically developed. The ADVOS hemodialysis system seeks to restore the homeostasis of the patient through a multiorgan approach by efficiently removing water-soluble and protein-bound toxins, and by improving the blood composition through acidosis correction and CO_2_ removal [8–13]. Therefore, clinical data are needed to demonstrate the impact of IL-6 removal with ADVOS multi in the outcome of critically ill patients with multiple organ failure. Furthermore, the consequences that IL-6 removal and a possible concomitant depletion of molecules of similar characteristics through extracorporeal blood purification is still a matter of debate [39]. Thus, we suggest monitoring the clinical course of IL-6 in patients treated with ADVOS and the inclusion of the routine measurement of this cytokine as an endpoint in future clinical trials.

### Limitations

Our study is limited by it is an in vitro nature and its applicability to the clinical setting might be restricted. Additionally, the results are based on short experiments that do not reflect the expected duration of a treatment. However, we have used a well-established blood model that allows to establish proof-of-concept data for the removal of relevant toxins, as shown previously [13, 40]. Furthermore, the design of the experiments with multiple sample extraction during the first hours allows a reliable estimation of the expected removal of IL-6.

## Conclusion

In summary, our study demonstrates the efficient and continuous removal of IL-6 by ADVOS multi, even at low blood flow rates, as evidenced in vitro. We observed concentration-dependent removal within the initial timepoints, followed by a more consistent elimination in the subsequent hours. Recirculation of the dialysate proved effective in preventing the removal of low IL-6 concentrations. However, further investigation, both in vitro and in clinical settings, is essential to gather comprehensive and meaningful data.

## Data Availability

The datasets used and/or analyzed during the current study are available from the corresponding author on reasonable request.

## Abbreviations

ACLF: Acute-on-chronic liver failure
ADVOS: Advanced organ support system
EBP: Extracorporeal blood purification
HCO: High-cut-off
IL-6: Interleukin-6
MCO: Medium-cut-off
